# “It's the Wild West Out There”: A Qualitative Study of the Views and Preparedness of Health Professionals in Helping Young Adult E-cigarette Users to Quit

**DOI:** 10.1093/ntr/ntae117

**Published:** 2024-05-24

**Authors:** Nicola Rahman, Bernadette Sebar, Ernesta Sofija

**Affiliations:** School of Medicine and Dentistry (Public Health), Griffith University, Gold Coast, QLD 4215 | G01_3.30, Australia; School of Medicine and Dentistry (Public Health), Griffith University, Gold Coast, QLD 4215 | G01_3.30, Australia; School of Medicine and Dentistry (Public Health), Griffith University, Gold Coast, QLD 4215 | G01_3.30, Australia

## Abstract

**Introduction:**

Young adults (YA) are attempting to quit vaping, with many accessing smoking cessation programs with a lack of reported efficacy, highlighting the need for targeted vaping cessation support. Young people report seeing health professionals (HPs) as potential sources of support in the quitting process. Additionally, the current changing regulatory landscape around vaping in Australia potentially increases the number of those seeking health professional help for cessation. However, limited research exists on HPs’ views and preparedness to assist YA with their vaping cessation; thus, this exploratory study aimed to gain insights into their readiness to support YA in quitting vaping.

**Aims and Methods:**

Data were gathered via eight co-design workshops (two groups each of two hours duration and six semi-structured interviews of 1-hour duration), facilitated online with 12 HPs. Data underwent thematic analysis.

**Results:**

HPs expressed a need for more information in supporting YA to quit vaping, with them presently relying on informal pathways of support and information for their practice. Participants reported a lack of evidence-based guidelines and a reluctance to prescribe nicotine vapes, expressing conflict with the changing regulatory landscape in Australia.

**Conclusions:**

Our findings identify a significant gap in health professional preparedness in supporting vaping cessation. HPs are working within a rapidly evolving regulatory environment and are feeling unprepared to address the widely spread issue of vaping, especially among young people. We demonstrate the critical need for guidelines and training of HPs to enable them to better support young people in quitting vaping.

**Implications:**

This qualitative study offers unique insights into the views and readiness of Australian HPs to support young people to quit vaping, specifically in the context of recent regulatory reforms. The results highlight the need for evidence-based guidance and training for HPs to inform their vaping cessation support practice.

## Introduction

The significant growth in young people using e-cigarettes, or vaping, is a critical global public health issue.^1–3^ Of particular concern is vaping among youth and young adults (YA),^4,5^ a population more vulnerable to the health risks associated with nicotine use during this period of physical development and brain maturation.^6^ In Australia the highest current use is amongst 18–24 year olds,^7^ with a 3-month prevalence of 19.8% between January and March 2023. YA have often never smoked before trying e-cigarettes and are at a three-fold increased risk of initiating subsequent combustible cigarette smoking.^8^

Increasing vaping prevalence is of concern given the growing body of evidence highlighting the health risks of e-cigarette use,^8^ in particular, the risk of nicotine dependence.^8^ This dependence is exacerbated in e-cigarette use because of the varying nicotine concentrations, ease of high nicotine consumption, and greater social acceptance of vaping among youth.^9^ Global evidence demonstrates e-cigarette users wish to quit,^10,11^ or have made quit attempts,^12,13^ with many accessing tobacco smoking cessation services as a means of support, yet with limited success.^14–16^ This highlights the need for targeted vaping cessation support.

Currently, vaping cessation programs are predominantly based on models for combustible cigarette smoking and lack evidence-based strategies specific to the nuances of e-cigarette use.^17–19^ Quitline services based on smoking cessation protocols have demonstrated success with people who either exclusively smoke or vape,^20^ however further age-related evaluation is required to understand the efficacy in YA populations. The Royal Australian College of Practitioners (RACGP)^21^ acknowledges a lack of evidence-based vaping cessation strategies available to health professionals (HPs) who may be involved in caring for young people in this space. This is concerning given that YAs who vape report thinking a HP would be able to help them quit,^14,22^ with young people who vape and also smoke are more likely to seek help from a healthcare provider.^23^ In addition, recent regulatory reforms by the Australian Government on the importation, manufacturing, and supply of vapes are designed to protect young people from the risks of vaping.^24^ Importation of disposable vapes regardless of nicotine content is banned, and access to supplies via the Personal Importation Scheme is now closed.^24^ As of January 1, 2024, a prescription is required to lawfully access therapeutic vape products for either smoking cessation purposes or managing nicotine dependence, which is dispensed via a pharmacy.^24^ The RACGP advises this is likely to result in increased numbers of YA seeking the support of a HP to manage their nicotine addiction, as reported by the Department of Health and Aged Care [DoHAC] impact analysis.^25^ This pinpoints the importance of exploring the views of HPs to facilitate our understanding of their readiness to support young people in this space.

Research examining the preparedness of HPs in supporting people to quit e-cigarettes is sparce, with studies focusing on the communication around the use of e-cigarettes^26^ and training needs of HPs in U.S. populations.^27^ A systematic review of HPs’ beliefs and self-reported practices towards e-cigarettes and their use in smoking cessation, illustrated HPs’ mixed views of their efficacy and health risks.^28^ This review called for further research in countries with differing regulatory contexts, such as Australia, and recognized the need for data from additional practitioners such as pharmacists and nurses, acknowledging their important role in this space. The impact of Australia’s regulatory model on HPs smoking cessation practices was recently explored, with participants identifying limitations of the current system, such as difficulty accessing safe products for smoking cessation purposes and a reluctance to prescribe e-cigarettes due to concerns for ongoing nicotine dependency.^29^ There is a need for further qualitative exploration of HPs’ thoughts and views on their own readiness for vaping cessation practices. To address this gap, the current paper explores if HPs felt equipped to support young people to quit vaping.

## Materials and Methods

The results reported in this paper are drawn from a larger two-phase study co-designing a vaping cessation program for YA. Phase one of this study consisted of an online survey of YA who currently or used to vape, examining their patterns of vaping, their quit intentions and quit attempts, reported elsewhere.^13^ The current paper is part of phase two, consisting of co-design workshops and semi-structured interviews for individuals to further explore vaping cessation and the insights of YA who currently or used to vape and HPs as key stakeholders. The focus of this paper is the views and perspectives of HPs.

### Study Design

Co-design workshops and semi-structured interviews were employed using Trischler et al.’s^30^ seven-step framework; co-design methodologies have demonstrated success in gathering in-depth qualitative insights of service users and providers for public health interventions.^30^ The workshops and interviews explored the knowledge, experiences, and views of HPs around supporting vaping cessation of YA in Australia. The co-design framework and its application in this study to both group and individual sessions are detailed in Supplementary Appendix.

### Recruitment

The diverse choice of HPs was informed by the phase one survey question asking YA’s who currently or used to vape, who they would or had approached for support to quit vaping. Recruitment methods included a volunteer for research broadcast via the university, a local primary care research network, LinkedIn, and the research team’s professional networks. All HPs who responded were accepted for data collection. This included representation of metropolitan and regional locations.

### Data Collection

To accommodate the availability and geographic spread of multiple HPs, the workshops were held online via Microsoft Teams. Each session commenced with thought-provoking questions, as part of the sensitization step of co-design, to brainstorm the topic and open the discussion around vaping cessation: “what do you know about vaping cessation?” followed by an explorative open discussion. This was followed by other prompts such as “what processes do you follow?,” “why might young people find it hard to quit?” and “who do you think YA might approach for support?.” Discussion was allowed to flow organically to enable exploration of the topic.

### Data Analysis

Workshops were audio recorded, transcribed verbatim, and analyzed using reflexive thematic analysis.^31–33^ Coding, using NVivo software, was inductive, iterative, and without a structured code book.^32^ This ensured the interpretation of meaning was data-driven. Additionally, deductive analysis was utilized to ensure the coding and theme development were meaningful to the research questions. reflexive thematic analysis allowed the researchers to generate subjective themes of meaning, whilst reflecting on their experiences, understanding, and assumptions, and how these might shape the coding and theme development. The concepts were then carefully and thoughtfully organized into an overarching narrative. The steps for this reflexive thematic analysis included data familiarization (NR), identifying codes within the transcripts (NR), collating the codes into potential themes (NR), reviewing, developing, and naming themes (NR, BS, and ES), and reporting the themes (NR, BS, and ES). The research team met regularly during this analytic process to ensure agreement on the final themes.

### Ethical Approval

Ethical approval was granted by the university’s Human Research Ethics Committee (GU Ref 2020/ 925), with all participants providing voluntary signed informed consent.

## Results

### Participant Characteristics

Data collection took place across two group workshops, and six individual sessions held between November 2023 to January 2024. The allocation of HPs to sessions was based on their availability, which required flexible scheduling. Twelve HPs were engaged in total, with sessions taking between one to two hours. Group one consisted of an Addiction Psychiatrist and Mental Health Educator. Group 2 included a Health and Well-being Counselor, Health Promotion Officer, Nurse, and Pharmacist and the remaining participants were individual sessions. Participant professions are listed in Table 1; the provision of specific geographic locations has intentionally remained broad to maintain confidentiality.

**Table 1.** Participant Discipline, Location, and Allocated Pseudonym

Participant discipline	Practice type	Geographical location	Allocated code name
General Practitioner	Community Health Center	Regional South Australia	GP1
General Practitioner	Community Health Center	Regional Queensland	GP2
General Practitioner	Community Health Center	Brisbane Queensland	GP3
Health and Well-Being Counselor	Community Health Center	Gold Coast Queensland	Counselor
Mental Health Educator	Statewide Program	Gold Coast Queensland	MH Educator
Pharmacist	Community Health Center	Melbourne Victoria	Pharmacist
Clinic Nurse	Community Health Center	Gold Coast Queensland	Nurse
Addiction Psychiatrist^*^	Drug & Alcohol Use Facility, Private Clinic/ Consultation	Brisbane Queensland	Psychiatrist
Drug and Alcohol Educator	National Audience	Sydney New South Wales	DA Educator
Health Promotion Officer	Community Health Center	Regional Victoria	HP Officer
Psychologist	Community—Consultancy	Gold Coast Queensland	Psychologist
Psychologist	Private Practice	Melbourne Victoria	Psychologist2

^*^TGA Authorized Prescriber at the time of data collection.

### Thematic Analysis

The analysis generated 18 codes (NR), which contributed to the development of four main themes (NR, BS, ES) each reflecting a centrally organizing concept of related meaning. The themes describe HPs (1) knowledge of vaping cessation, (2) a lack of evidence-based information, (3) reluctance in changing regulatory landscape, and (4) adapting health assessments. All HPs expressed feelings of unpreparedness in practice and a lack of guidance or support informing them of best practices.

#### Theme 1: Knowledge of Vaping Cessation

Participants quantified their knowledge of vaping cessation in terms of experience to date in supporting anyone through this process. The Psychologist had previously supported individuals through smoking and cannabis cessation, but not vaping cessation: “No, that’s a whole new ball game and I haven’t actually had any patients who’ve approached me....” Similarly, the general practitioners (GPs) cited limited experience, despite knowing some of their patients vaped, with GP1 saying “Obviously I haven’t faced anything like that, that someone has walked in and asked ‘OK, I want to quit vaping, I need your help’.” *This was reiterated by GP2, who said* “Personally, I don’t have many people that have ever come to me saying ‘I really want to quit vaping’.”

The anticipated increased numbers of people who vape approaching GPs for prescriptions, because of recent regulatory changes banning the importation of disposable vapes^25^ had not yet been experienced in practice:

*“We’ve had no one coming for scripts this year...I mean it’s only been 10 days of the year I suppose but that’s what the GPs were saying, well from 1* ^*st*^ *January everyone will be flooding in. They’re not flooding in*” (GP3).

Knowledge of vaping cessation was also described by the Pharmacist according to limited experience, who felt their practice was influenced by the lack of current GP prescriptions:

“*What I’m supposed to see is a prescription written out as nicotine vaping liquid and as a pharmacy supplying that vaping liquid together with the vaping device medically approved by the TGA...so far in maybe perhaps the whole year... I have not seen the GPs that I’ve worked with prescribe any of these products”*.

This lack of prescription by GPs was described as having a ‘knock-on’ effect on pharmacists: “Because they have not received a script... and they do not know where to buy what, who are the wholesalers to purchase these products” (Pharmacist).

The participants also willingly disclosed that there were a lot of “unknowns” about vaping cessation; as evident in GP2’s comments “There’s a lot of unknowns about the chemical makeup of the various vapes” and “I guess we don’t really know the long term harm apart from the nicotine component...maybe mobile phones are going to do worse damage to us in the longer term.” Additionally, the lack of long-term data on the consequences of vaping meant that one participant felt their vaping cessation advice would not have the desired effect: “There’s a bit of fear mongering on our sides... it actually causes more confusion... with confusion, people just throw their hands up and go, I don’t know, I don’t care” (Counselor).

Participants reported their vaping cessation knowledge by identifying differences between vaping and smoking cessation, such as “the smell, the antisocial kind of side to cigarette smoking is not there for vaping” (GP3); with this lack of associated stigma causing “much less motivation to quit vaping” (GP3). It was expressed by participants that due to the high levels of nicotine in vapes, “the nicotine addiction is possibly stronger” (GP3) and measurement of nicotine consumption was harder, making cessation advice challenging:

*“When it comes to nicotine and how much they’re actually inhaling, how do you measure it? Do they know how much they’re actually inhaling?...when you ask someone if they’re smoking, you say, how many and they’ll say three to five, or a pack a day... but* (with vapes) *how do you quantify the nicotine?”* (Nurse).

The same participant felt their knowledge of smoking cessation products was comprehensive compared to vaping and *“could list off all of the products that are available for smoking cessation”* but could not do the same for vaping cessation. Strategies that had previously been offered for smoking cessation purposes were identified as potentially less effective for vaping cessation due to the product differences:

“*So if they usually smoke a pack (of cigarettes)...so that you’ve budgeted for the day’s 17 cigarettes, and you work intrinsically to limit it... to 15 cigarettes and 13 cigarettes... but you can’t do that with vapes in the same way, because it’s not a kind of discreet amount...”* (Psychiatrist).

One GP perceived their role in supporting YA who vape to quit to be viewed negatively by their patients: “We are sitting there as the boring fuddy duddy old GP who clearly knows nothing, saying you should quit” (GP3). This negative perception of a GPs knowledge was reflected by others:

*“If you said who has credibility, who would you go to? Credibility is always GPs, it’s right up the top every single time. Whenever you say that to doctors, they have a nervous breakdown because they know nothing about drugs”* (DA Educator).

This expression by the participants of their limited knowledge and experience of vaping cessation support practices appeared to be confounded by the lack of evidence-based information, as illustrated by the next theme.

#### Theme 2: Lack of Evidence-Based Information

A distinct theme was the lack of available evidence-based information on vaping cessation. Practitioners expressed a need for guidelines and articulated uncertainty when it came to prescribing nicotine vapes: “The only thing we need at this moment from a GP perspective is a very clear-cut guideline. If someone comes to me for vaping cessation or stopping vaping, what should I do, so these are the steps...” (GP1). This was reiterated by GP2, “I guess you just don’t really know the products... we don’t really have any formulas for working out like how many vapes a day equates to what level of nicotine replacement.” Participants were concerned their lack of informed action may even make matters worse if they just “whack the full-strength patch on” and “if that was exceeding what your previous nicotine consumption was... you could make them worse” (GP2).

GP3 questioned how they were supposed to prescribe nicotine vapes, in the absence of specific guidelines: “There’s certainly no prescription guidelines... we have a mock patient in our software... I could show you what is in there for how many nicotine products I can prescribe, but I look at them and just think I have no idea” (GP3). This is complicated by the lack of clear labeling on products and the lack of prescriber information usually provided by the Monthly Index of Medical Specialities. The Pharmacist suggested HPs are looking to each other for guidance: “You could say the doctors need advice from the pharmacies... what to prescribe, but then the pharmacist would say we do not know because you are the prescriber....”

The GPs expressed the need for prescriber training as part of the guidance update, saying “The questions have been, where can I get training on this?” (GP3) and “I know the RACGP say they’ve got a new module coming out, they keep saying it’s coming out, but it hasn’t come out yet” (GP2).

The need for guidelines was also evident in the response by the Nurse, who felt it would be useful to know “the right questions to ask... having more of a process as to how to best support them,” indicating the need for vaping cessation guidance as not solely product or prescription-related. This participant discussed how they follow smoking cessation practices, but that for vaping “there’s no real guidance, procedure or policy.”

The lack of evidence-based information was also demonstrated by participants expressing the desire for helpful sources to guide their vaping cessation support practice. GP1 commented they would look to the RACGP for therapeutic guidelines or “check whether they have published any article on vaping cessation,” while GP3 was waiting for “an RACGP education webinar about the new prescribing and regulations.” Overseas sources were relied upon in the absence of Australian-based guidelines such as “The Cleveland Clinic” (GP1), and informal sources such as social media platforms: “There’s massive GP information on Facebook forums...vaping/ e-cigarettes always comes up... From January 1st there’s been a bit more chatter in GP forums” (GP3). Independent organizations previously providing smoking cessation guidance, such as the Primary Healthcare Network or the National Prescription Scheme were no longer considered helpful. Similarly, help from colleagues such as educational Grand rounds or journal clubs had not yet covered vaping cessation, due to an apparent lack of specialist information: “It’s in the too hard basket, I think” (GP3).

#### Theme 3: Reluctance in Changing Regulatory Landscape

The code of reluctant prescribers contributed to the theme of a changing regulatory landscape. HPs expressed apprehension at prescribing nicotine vapes with concerns about the consequences:

“*When we think of smoking cessation... we are not replacing it with anything else, so if we replace it with vaping... if we want to try stopping vaping, then they will probably end up getting back to smoking cigarettes”* (GP1).

Reluctance to prescribe nicotine vapes, for fear of the patient’s inability to quit them was strongly expressed by GP3: “I would never suggest vaping as a quitting smoking tool myself... I don’t see any role for suggesting a smokers 18 to 24 would move on to vapes... I think we would never get them off vapes.” The view that vapes were “less bad” than cigarettes was provided as justification for prescribing unflavored nicotine as “presumed harm minimization in people who have tried...everything” although this prescriber also said, “I don’t find vaping as an effective way of assisting people to stop nicotine use” (Psychiatrist).

The proposed regulatory changes from January 1, 2024 to facilitate GPs and Nurse Practitioners to sign up to the Special Access Scheme C to prescribe therapeutic vaping substances,^21^ was met with further reluctance:

*“Not many of us are interested into going anywhere to tick that box (to prescribe)... initially when it first came out, this was going to be great harm minimisation... then in the last couple of years, seeing no one who goes on vapes quit smoking... vapes do heaps of harm... huge amounts of nicotine... hugely addictive... That’s not where we thought this thing would land” (GP3).*

One participant felt that this changing landscape needed more time for the familiarity of processes to be accomplished, comparing it to the cannabis prescribing context, *“Doctors were not very confident, but now in about 2 years... doctors are very confident”* (Pharmacist). The changing landscape also needed more action, ***“****I totally agree that more education on this is absolutely necessary”* (Counselor). There was also an expression of conflict with the regulations, concerned they may discourage YA from disclosing their vaping and exacerbate illegal vape production:

*“You get these backyard people making up vapes and you know, pouring a bit of rotten watermelon juice in there with the nicotine crushed up and just selling out there... it’s a bit of a Wild West sort of situation... I’m just not sure the government really is doing the right thing necessarily by just banning it completely”* (GP2).

The participants struggled to understand how illegal vape sales continued, despite the prohibitory regulations on sales: “Vape stores must have a huge stockpile that they are still selling illegally” (GP3) and “I don’t actually understand how, it’s supposed to be so regulated. How is it possible and so unregulated at the same time?” (Counselor).

#### Theme 4: Adapting Health Assessments

When discussing how patient’s vaping was assessed, participants cited how the opportunity may not be appropriate or relevant when YA presents to their service: “Mostly from that age group like 15 to 25, either they have broken their bones, or they have got mental health issues” (GP1) and “we don’t check anyone before 40 unless they have got a very strong chance of having a heart disease... ” (GP2). The assessment tended to be determined by age: “It’s more the older people with chronic diseases that get our comprehensive assessment with all this sort of drug and alcohol check...” (GP2). The onus of assessing vaping-related health appeared to be passed on to the patient, with GP2 reporting that young people are “just not really interested in it or motivated to want to come and do that.”

However, where vaping behaviors were assessed, both the GPs and the Nurse reported their current patient information systems did not yet include specific vaping-related questions: “We don’t have any questions related to vaping” (GP1) and “It’s not even an option in our drop down box of, you know are you a nonsmoker or ex-smoker” (Nurse). Participants cited resorting to adapting their patient data input using the smoking questions:

*“We can’t put vaping in our software. There’s tick boxes - How many cigarettes per day?... Not vaping... You have to retype it because it’s for cigarettes. It’s 1 to 9 cigarettes, greater than nine, greater than 20...”* (GP3).

Alternative health assessment tools were also considered as a way of discussing the drug and cigarette context, with GP3 citing the HEADSS adolescent psychosocial assessment tool^34^ for the inclusion of vaping. However, this was dependent on the individual situation and not a routinely used tool:

*“I probably still would use that HEADSS questionnaire on someone depending on their personality, up to 21, that kind of thing. It’s not just for the under eighteens, it gives us that whole home environment, employment... what their whole context is... drugs and cigarettes” (GP3).*

## Discussion

YA who vape perceive HPs as suitably placed to support them in quitting.^22,23^ However, the current study highlights HPs lack of preparedness in assisting those who vape to quit. To the author’s best knowledge, this is the first study to explore the views and readiness of HPs in Australia since the announcement of proposed regulatory reforms in May 2023.^35^ Previous research has examined the views of HPs towards e-cigarettes in supporting smoking cessation and in different regulatory contexts.^26,28,36^ This study broadens our understanding of the views of HPs, by focusing on vaping cessation support practices. The inclusion of additional professionals considered important in the support of young people’s vaping cessation, adds to the limited qualitative data gathered in this area. Furthermore, whilst the training needs of HPs supporting vaping cessation have previously been explored in the United States,^27^ this study adds further nuance to our understanding of professionals’ needs in the precautionary regulatory landscape of Australia.

First, HPs describe their knowledge of vaping cessation in terms of having limited experience, and being unfamiliar with processes and products. The participants expressed a strong desire for evidence-based information, and more specifically, guidelines on vaping cessation to inform their practice. They reported “unknowns” surrounding how to prescribe nicotine vapes, the lack of data on long-term health risks, and concerns for continued nicotine dependence; leading to a reluctance to prescribe under the new Authorized Prescriber Scheme.^24^ Currently the RACGP^21^ acknowledges the limited research on pharmacotherapy and vaping cessation interventions, with the recent guidance update referring to a pharmacist-assisted program paired with behavioral counseling from a study based in the United States.^37^ The guidance also refers to smoking cessation support such as nicotine replacement therapy but advises that current nicotine replacement therapy is not TGA-approved or subsidized for vaping cessation purposes.^21^ This lack of clear guidance on best practices may contribute to the reluctance of practitioners to prescribe. HPs find themselves in a changing regulatory landscape for which they are ill-prepared, highlighting the need for urgent action in the provision of evidence-based information and training.

Second, our findings confirm there are differences in supporting vaping cessation, compared to smoking cessation, as cited in the perceptions of the participants and previously suggested by young people who used to vape.^22^ Advice on how to prescribe nicotine has been provided to GPs, in the context of harm-minimization and smoking cessation practices.^38^ Nevertheless, previous research examining the knowledge of HPs when prescribing e-cigarettes for smoking cessation found many HPs were skeptical about the role of e-cigarettes for this purpose, with concerns about a potential new addiction.^28^ We have recently seen an explosion of e-cigarette use among young people, triggering regulatory changes in Australia, designed to enforce users to access nicotine vapes via their GP or prescribing nurse practitioner.^24^ As revealed by our study’s participants, the often unknown or high amounts of nicotine in many products make the process of prescribing nicotine vapes challenging. Previous examination of submissions (*n* = 1405) by self-reported e-cigarette users to a TGA consultation found that 172 (12%) believed HPs are unwilling and inadequately trained to prescribe nicotine vapes.^39^ Additionally, 674 (48%) submissions reported smoking relapse as a potential negative consequence of the prescription-only model.^39^ The resulting juxtaposition of reluctant prescribers against a backdrop of changing access to nicotine vapes may result in users turning to other sources of nicotine, such as cigarettes, a concern described by those who vape. Urgent action is needed to provide evidence-based vaping cessation guidelines that include specific information addressing nicotine dependence and its safe reduction via the prescription model.

Third, our findings demonstrate the need for a collaborative approach between allied HPs in providing vaping cessation support across the primary care setting. Demonstrated successful outcomes of a pharmacist-led vaping cessation pilot study in the United States^40^ highlight the potential value in multiple pathways of support by HPs. HPs involved in the care of young people (eg, counselors and nurses or psychologists), cited the requirement for informed strategies on how to broach the opportunity of vaping cessation, like questions to ask and processes to follow. The requirement for training and skills for HPs supporting vaping cessation was recently identified in U.S. professionals, with the need for training activities to address specific work settings.^27^ The outcome of the study was the development of a case-based educational webinar, informing HPs how to support young people to quit vaping, with the users reporting an improvement in their knowledge and competence.^27^ The current study demonstrates the need for similar training in Australia, to up-skill practitioners in the provision of vaping cessation support. There is also the need for updated digital patient management systems to streamline related health assessments.

### Limitations and Future Directions

The study has some limitations that should be acknowledged. The 12 participants may not represent the views of HPs across Australia; differences in state allocation of resources for public health and primary care, made more complex by population demographics and needs, may influence findings. Further research to examine the needs of HPs on a larger scale is also required to inform the preparation of education and training programs for supporting YA in quitting vaping. Nevertheless, the engagement with the 12 HPs in exploring vaping cessation provides valuable insight into the current climate of practitioner readiness.

## Supplementary material

Supplementary material is available at *Nicotine and Tobacco Research* online.

ntae117_suppl_Supplementary

## Data Availability

Data from this qualitative study are not publicly available to minimize risk from deductive disclosure and in the interests of protecting the professional occupations of participants.
