# Supplementary material for: “It's the Wild West Out There”: A Qualitative Study of the Views and Preparedness of Health Professionals in Helping Young Adult E-cigarette Users to Quit
Source: Nicotine Tob Res. 2024 May 24;26(11):1538–44. doi: 10.1093/ntr/ntae117 (PMC11494468; doi:10.1093/ntr/ntae117)
Supplement: ntae117_suppl_Supplementary [file ntae117_suppl_supplementary.docx]

**APPENDIX**

**Summary of the 7-step co-design framework in action.**

| **Co-design Stage** | **Purpose and Application** |
| --- | --- |
| *Steps 1-3 occur prior to the workshops and semi-structured interviews in an iterative cycle.* | |
| 1) Resourcing | A review of the literature informed the team of the research topic insights and study design, with identification of key input for workshops. This step helps the research team understand the underlying issue to be addressed. |
| 2) Planning | Regular meetings of the research team to plan each stage of the data collection including; organising the workshop modality, how data would be collected, who would facilitate the workshops, and discussion of contingency planning for any unexpected occurrences such as technical issues. A Padlet virtual notice board was created to provide visual prompts for each question/activity. |
| 3) Recruiting | Recruitment of health professionals [HPs] was informed by phase one of the research (see section 2 of text). A diverse range of mediums were used to recruit participants in an iterative process to maximise possible participation of HPs. Informed consent is ensured. HPs were offered a $50 digital gift voucher as a token of appreciation for their participation. |
| 4) Sensitisation | This step aims to engage participants with the use of activities, facilitating their reflection on the underlying topic. Sensitisation may be held prior to workshops/interviews, for example with the use of a questionnaire. However, due to the time constraints of participants this was held during the workshops/ semi-structured interviews. |
| 5) Facilitation | ***Introduction:*** Use of warm-up activities such as participant introductions, testing cameras and microphone in Teams to prepare and an explanation of how the workshop will run.  ***Sensitisation*** ***activities*:**  a) Thought provoking questions designed to elicit knowledge and insights of participants:  - What do you know about vaping cessation?  - Who do you think young adults might approach for support?  - Why might young people find it hard to quit?  b) Use of ‘ideas cards’ representing current vaping cessation strategies used based on the literature review ‘likes/ dislikes/ improvements/ better idea’ feedback technique applied to each idea.  ***Design ideation:*** participants discuss their ideal vaping cessation support, encouraged to develop strategies of ideas cards or generate new ideas. Reporting back via recorded discussion and written notes. |
| *Steps 6 and 7 occurred after workshop completion.* | |
| 6) Reflecting | Young adult [YA] insights from phase one of study shared with key stakeholders as part of the iterative co-design process and subsequent workshops. All qualitative data from YA and key stakeholder workshop and interviews are analysed, including written notes and discussion recordings, based on Braun & Clarke’s Reflexive Thematic Analysis (2021). Coding and theme development was conducted by research team. |
| 7) Building for change | The final step is ongoing and will form the basis of the next study to ‘realise’ the design informed conceptual model, in partnership with designers, external organisations and service delivery experts. |
